# Phosphoproteomic landscape of pseudorabies virus infection reveals multiple potential antiviral targets

**DOI:** 10.1128/spectrum.03010-23

**Published:** 2023-11-22

**Authors:** Zongyi Bo, Xiaojuan Li, Chengcheng Zhang, Mengjiao Guo, Yongzhong Cao, Xiaorong Zhang, Yantao Wu

**Affiliations:** 1 Joint International Research Laboratory of Agriculture and Agri-Product Safety, The Ministry of Education of China, Yangzhou University, Yangzhou, Jiangsu, China; 2 Jiangsu Co-Innovation Center for the Prevention and Control of Important Animal Infectious Disease and Zoonoses, College of Veterinary Medicine, Yangzhou University, Yangzhou, Jiangsu, China; University of Wisconsin-Madison, Madison, Wisconsin, USA

**Keywords:** pseudorabies virus, phosphoproteomic landscape, virus-host interaction, antiviral targets

## Abstract

Phosphorylation, a significant posttranslational modification, is implicated in nearly two-thirds of all proteins. The involvement of phosphorylation in various cellular responses is known to play a crucial role in the replication of pseudorabies virus (PRV). However, the phosphoproteomic landscape during PRV infection remains unknown. To address this, a tandem mass tag-based liquid chromatography-tandem mass spectrometry was performed. It was found that a total of 7,342 phosphosites were detected; among these, 1,197 phosphosites were significantly upregulated, and 1,120 phosphosites were significantly downregulated when the threshold was set at 1.5. Gene ontology analysis of these significantly changed proteins showed that they mainly participated in mRNA processing, RNA splicing, stress granule assembly, and other pathways. Kyoto Encyclopedia of Genes and Genomes pathway enrichment analysis showed that these proteins were associated with cell cycle, spliceosome, tight junction, mRNA surveillance, and other pathways. Additionally, an immunoblotting assay confirmed the phosphoproteomic results by detecting the protein level of several upregulated phosphorylated proteins, which included pBRAF, pRPS6KA1, and pSTMN1. Further experiments using siRNA showed that knockdown of BRAF, RPS6KA1, and STMN1 significantly inhibited the replication of PRV. In summary, this study provides insight into the phosphoproteomic landscape during PRV infection and contributes to a better understanding of the mechanisms underlying PRV replication.

**IMPORTANCE:**

Pseudorabies virus (PRV) is a kind of alpha herpesvirus that infects a wide range of animals and even human beings. Therefore, it is important to explore the mechanisms behind PRV replication and pathogenesis. By conducting a tandem mass tag-based phosphoproteome, this study revealed the phosphorylated proteins and cellular response pathways involved in PRV infection. Findings from this study shed light on the relationship between the phosphorylated cellular proteins and PRV infection, as well as guiding the discovery of targets for the development of antiviral compounds against PRV.

## INTRODUCTION

Pseudorabies virus (PRV), the only herpesvirus of the swine, belongs to the family *Herpesviridae*, subfamily *Alphaherpesvirinae*, which also includes herpes simplex virus type 1, herpes simplex virus type 2, varicella-zoster virus, equine herpesvirus 1, Marek’s disease virus, feline herpesvirus, etc. (1 – 3). PRV is an enveloped dsDNA virus, with about 150 kb genome length (4). While pigs are the only natural reservoir for PRV, it can also infect a broad host range, including sheep, deer, cattle, mice, etc. (5). More importantly, since the first case was reported in 1914, many PRV-infected cases in humans have been reported (6, 7). Therefore, it is important to study the interaction between PRV infection and host cellular responses for the control of PRV.

Posttranslational modification (PTM) refers to the amino acid residues modification in proteins by some enzymes, which includes phosphorylation, ubiquitination, SUMOylation, acetylation, glycosylation, etc. (8, 9). Among multiple PTMs, phosphorylation plays an important role in multiple cellular functions, which include innate immunity, cell cycle regulation, cell proliferation, apoptosis, programmed cell death, and DNA damage response (8). It has been reported that nearly more than two-thirds of human proteins are regulated by phosphorylation (10, 11). Phosphorylation of protein is a reversible process, which is composed of multiple protein kinases and phosphatases (12).

The host cellular environment is important for the infection and proliferation of viruses, so multiple proteins involved in phosphorylation processes are easy to be the target of viruses. Multiple phosphorylation events have been reported during the infection of PRV. It has been found that PRV infection could inhibit the formation of stress granules to facilitate its replication by dephosphorylation of eIF2α (13). Besides, RIPK3 and MLKL are upregulated by PRV, which results in cell necroptosis (14). Meanwhile, it was also found that PRV infection could trigger the activation of DNA damage response which was indicated by the upregulation of γH2AX (15). Therefore, it is speculated that the phosphorylation process plays an important role in the replication of PRV.

As an alpha herpesvirus, PRV itself can encode two viral protein kinases, UL13 and US3, which also play an important role in multiple host cellular responses (16). Previously, we have found that IRF3, an important transcription factor that participates in the production of interferon, is phosphorylated by PRV UL13 protein kinase, resulting in the inhibition of IFN-β (17). Additionally, we also found that γH2AX, the marker of DNA damage response, can also be upregulated by PRV UL13 protein kinase to promote viral proliferation (18). What is more, it was also reported PRV US3 protein kinase could promote retrograde transport in axons via activation of PI3K/Akt-mTORC1 pathway (19). PRV US3 could suppress the apoptosis by activating PI3K/Akt and NF-κB pathway (20). Except for the proteins phosphorylated by PRV-encoded two kinases, the other RV-encoded proteins can also participate in the phosphorylation events to regulate the cellular responses. For example, PRV IE180 protein could suppress the phosphorylation of translation initiation factor eIF2α (21). Collectively, these reports demonstrated that phosphorylation events play an important role in proliferation and pathogenesis of PRV.

In the present research, a quantitative phosphoproteome was conducted to explore the phosphorylation changes and host cellular responses upon PRV infection. It was found there were 2,317 significantly changed phosphosites upon PRV infection in PK15 cells; among these, 1,197 were upregulated, and 1,120 were downregulated. Besides, the characteristics of the phosphorylated proteins were analyzed, including molecular distribution, percentage of the phosphorylated (STY) sites, and phosphorylated motifs. Meanwhile, the gene ontology (GO) and Kyoto Encyclopedia of Genes and Genomes (KEGG) analyses of the significantly changed phosphoproteins were performed. Finally, three phosphorylated proteins, including B-Raf proto-oncogene, serine/threonine kinase (BRAF), ribosomal protein S6 kinase A1 (RPS6KA1), and stathmin 1 (STMN1), were chosen for analysis of their phosphorylation level and their effect on PRV replication, and the results showed that all these proteins were significantly upregulated, and all of them were necessary for efficient PRV replication. This research reveals the phosphorylation landscape of PRV infection, which significantly improves the study of the interaction between PRV infection and host cellular responses.

## RESULTS

### Sample collection of PRV-infected PK15 cells

**Fig 1:**
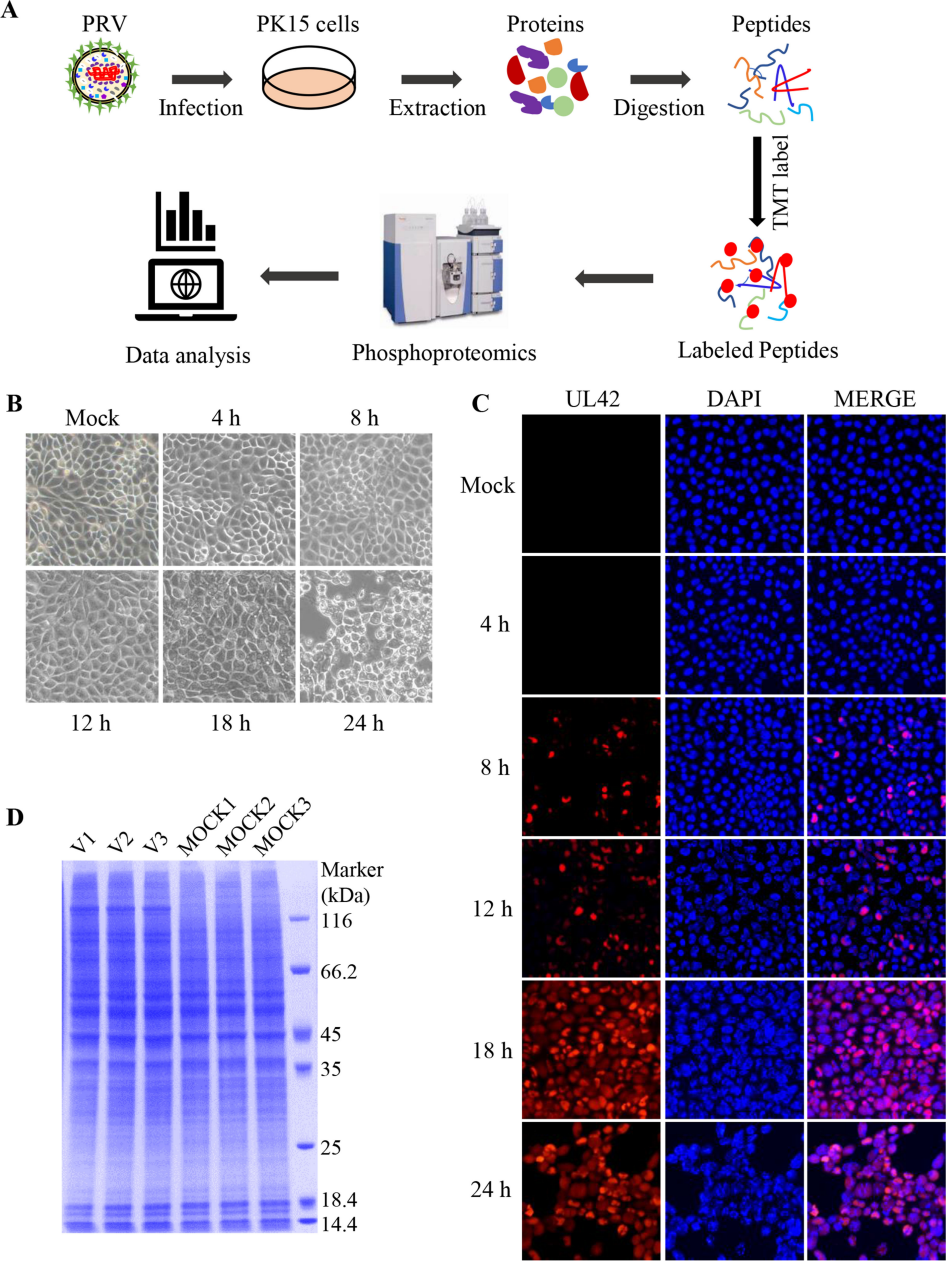
Workflow of quantitative phosphoproteomics analysis of PK15 cells infected with PRV. (**A**) Schematic representation of the experimental design. (**B**) Single-layer PK15 cells at 6-well plates were infected with JSY13 at an MOI of 0.5, and the morphological changes of the infected cells were captured at 4, 8, 12, 18, and 24 hpi. (**C**) PK15 cells in 6-well plates were infected with JSY13 (0.5 MOI) for 4, 8, 12, 18, and 24 hpi, then immunofluorescence staining was performed. (**D**) Each three 10× cm cell culture dishes were infected or mock infected with JSY13 (0.5 MOI), the cells were collected at 18 hpi, and equal amounts of the lysed samples were subjected into SDS-PAGE.

To identify the cellular proteins involved in the PRV replication, a quantitative phosphoproteomic analysis was conducted on PRV-infected PK15 cells as shown in Fig. 1A. First, the appropriate time points to collect the PRV-infected samples were determined. The PK15 cells in 6-well plates were infected with JSY13 (0.5 MOI), and the cell morphology was observed at 4, 8, 12, 18, and 24 hours post-infection (hpi). As shown in Fig. 1B, the results demonstrated that nearly most of the cells showed the cytopathic effect at 18 hpi. As time progressed, the infected cells seemed to detach and float in the medium at 24 hpi. Additionally, the indirect immunofluorescence assay (IFA) experiment was performed to confirm the status of viral infection, and it was found that nearly most of the cells were infected with PRV at 18 hpi (Fig. 1C). Next, the cells in six 10× cm cell culture dishes were infected or mock infected with JSY13 (0.5 MOI), and the cells were lysed and subjected to bicinchoninic acid (BCA) method for concentration determination. Then, equal amounts of the protein were subjected to SDS-PAGE to assess the quality of the harvested samples. The result of the SDS-PAGE showed that all samples had a good quality and met the requirement to be subjected to tandem mass tag (TMT)-based liquid chromatography-tandem mass spectrometry (LC-MS/MS) analysis (Fig. 1D).

### Landscape of phosphorylated proteins upon PRV infection

**Fig 2:**
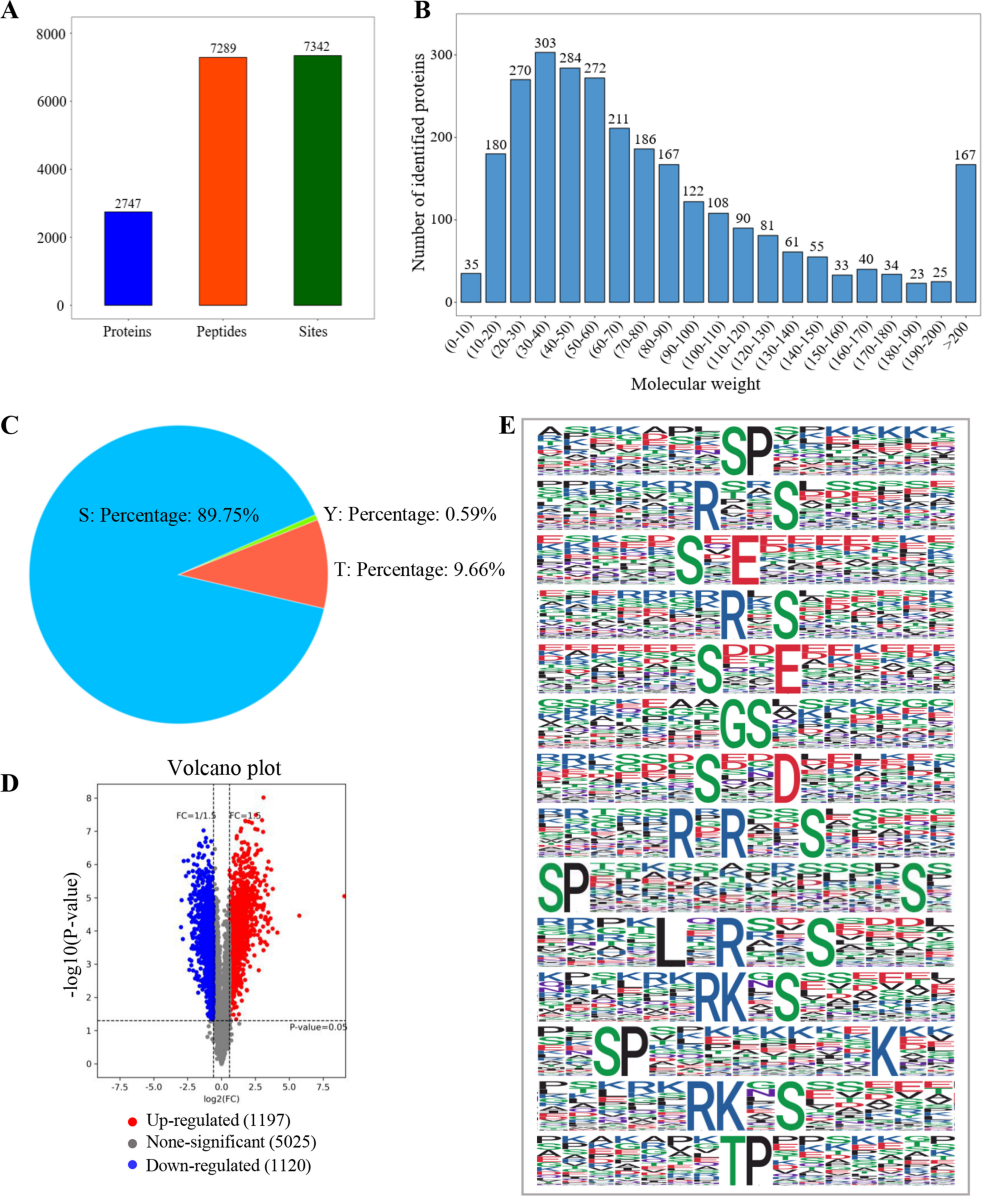
Landscape of phosphoproteome upon PRV infection. (**A**) Number of the collected phosphorylated proteins, peptides, and sites. (**B**) The molecular mass distribution map of phosphorylated proteins. (**C**) The percentage of the phospho (STY) sites. (**D**) Volcano plot analysis of the phosphosites when the threshold was set as 1.5. (**E**) The main phosphorylation motifs of the significantly changed phosphosites.

The same amount of the lysed peptides was labeled with TMT, and the enriched phosphopeptides were subjected to LC-MS/MS analysis. The results showed that there were a total of 2,747 phosphorylated proteins, 7,289 phosphorylated peptides, and 7,342 phosphorylated sites detected (Fig. 2A). The molecular distribution of these 2,747 proteins was also analyzed, the results showed that there were 303 proteins localized in 30–40 kDa, which accounted for the largest part of the phosphorylated proteins. With a rise in the protein molecule, there was a decrease in the detected phosphorylated protein (Fig. 2B). The phosphorylation sites usually happened at the residues of serine (S), threonine (T), and tyrosine (Y), and the percentage of phospho (STY) sites in a total of 7,342 phosphorylated sites showed that they were accounted for 89.75%, 9.66%, and 0.59%, respectively (Fig. 2C). Meanwhile, when the threshold of the significantly changed phosphorylated peptide was set as 1.5, the results showed that there were 1,197 significantly upregulated phosphosites and 1,120 significantly downregulated phosphosites, respectively (Fig. 2D). All of the collected phosphosites and the significantly changed phosphosites during PRV infection were listed in Table S1. The phosphorylation motifs, referring to the amino acids surrounding the phosphorylated sites, were also analyzed using the Motif-X. As shown in Fig. 2E, there were 13 serine motifs and just 1 threonine motif while no tyrosine motif in the top 14 screened phosphorylation motifs. Additionally, the results showed that Pro (P), Arg (R), and Glu (E) were the most abundant residues in the serine motif, while Pro (P) was the most abundant residue in the threonine motif. Except for host cellular proteins, the viral proteins may also be the target of phosphorylation. To explore whether there were phosphorylated PRV proteins during viral infection, the data were also aligned to sequences in PRV genome, and the result showed that there were a total of 50 viral proteins that were phosphorylated during the infection of PRV (Table S2).

### GO, KEGG pathway enrichment, and interaction network analyses of the significantly upregulated phosphoproteins

**Fig 3:**
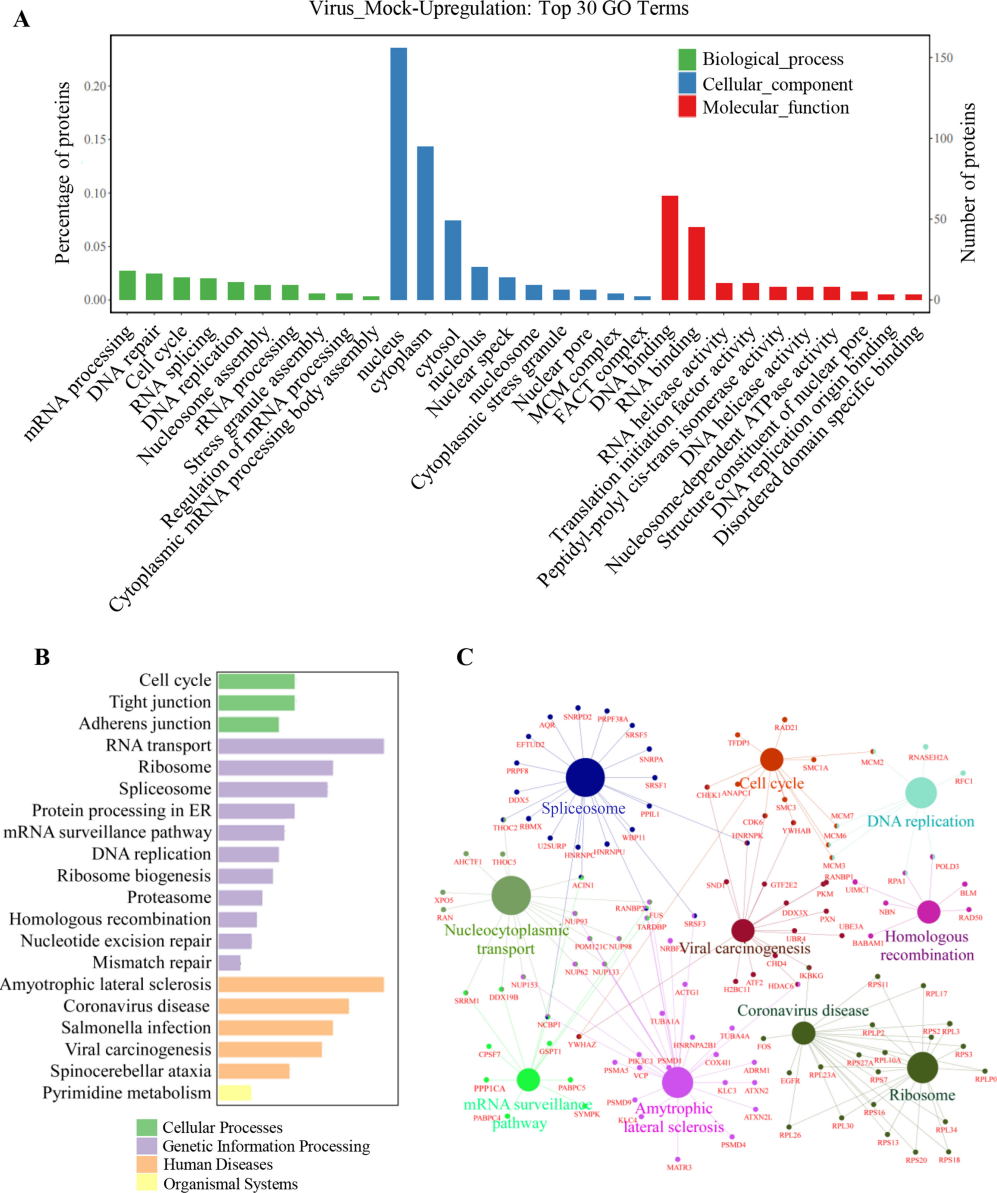
Gene ontology, KEGG pathway enrichment, and interaction network analysis of the significantly upregulated phosphoproteins. (**A**) GO analysis of the biological processes, cellular components, and molecular functions of upregulated phosphoproteins. (**B**) Top 20 enriched pathways using KEGG pathway analysis of the significantly upregulated phosphoprotein. (**C**) The main phosphorylated proteins in the top enriched cellular pathways and their interaction network analysis of the significantly upregulated phosphoproteins.

To investigate the protein sets of the 1,197 significantly upregulated proteins, the enrichment of them was analyzed based on the GO databases, which included the analysis of the biological processes, cellular components, and molecular functions (22 – 24). In terms of the biological processes, which refer to a biological objective to which the gene product contributes, the results of the top 10 terms included mRNA processing, DNA repair, cell cycle, RNA splicing, DNA replication, nucleosome assembly, rRNA processing, stress granule formation, and cytoplasmic mRNA processing body assembly (Fig. 3A). Regarding cellular components, which refer to the localization in the cell where the protein works, the results showed that the top 10 terms were nucleus, cytoplasm, cytosol, nucleolus, nuclear speck, nucleosome, cytoplasmic stress granule, nuclear pore, MCM complex, and FACT complex. In terms of the molecular functions, defined as the biochemical activity of a gene or its product, the results showed that the top 10 terms were DNA binding, RNA binding, RNA helicase activity, translation initiation factor activity, peptidyl-prolyl cis-trans isomerase activity, DNA helicase activity, nucleosome-dependent ATPase activity, structure constituent of nuclear pore, NDA replication origin binding, and disordered domain-specific binding.

In addition, to further understand the enrichment of signaling pathways of the significantly upregulated proteins, the Kyoto Encyclopedia of Genes and Genomes pathway enrichment was performed (25, 26). The results of the KEGG analysis showed that multiple kinds of pathways were regulated by PRV, including cell cycle, tight junction, adherens junction, RNA transport, ribosome, spliceosome, protein processing in endoplasmic reticulum (ER), etc. (Fig. 3B). Meanwhile, the protein-protein interactions involved in the several major enriched pathways were also analyzed as shown in Fig. 3C using STRING database and visualized by Cytoscape software. Collectively, these findings demonstrated that numerous phosphorylated proteins are upregulated during PRV infection, and they participate in multiple cellular processes, suggesting the significance of phosphorylation in PRV infection.

### GO, KEGG pathway enrichment, and interaction network analyses of the significantly downregulated phosphoproteins

**Fig 4:**
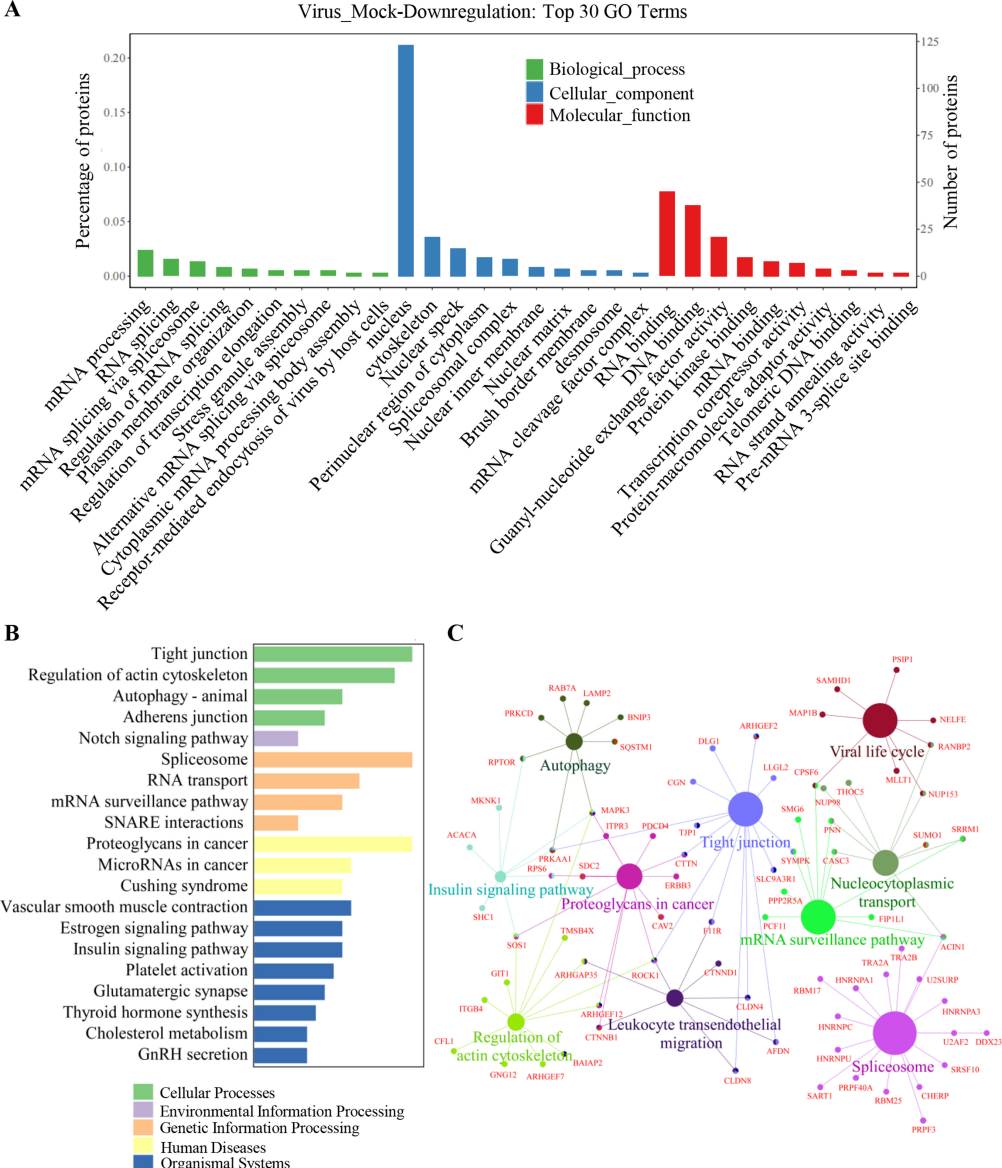
Gene ontology, KEGG pathway enrichment, and interaction network analysis of the significantly downregulated phosphoproteins. (**A**) GO analysis of the biological processes, cellular components, and molecular functions of the significantly downregulated phosphoproteins. (**B**) Top 20 enriched pathways using KEGG pathway analysis of the significantly downregulated phosphoproteins. (**C**) The main phosphorylated proteins in the top enriched pathways and their interaction network analysis of the significantly downregulated phosphoproteins.

The protein sets of the 1,120 significantly downregulated proteins were also analyzed based on the GO databases. The results revealed that the top 10 terms for biological processes included mRNA processing, RNA splicing, mRNA splicing via spliceosome, regulation of mRNA splicing, plasma membrane organization, regulation of transcription elongation, stress granule assembly, alternative mRNA splicing via spliceosome, cytoplasmic mRNA processing body assembly, and receptor-mediated endocytosis of virus by host cells (Fig. 4A). In terms of the cellular components, the top 10 terms included nucleus, cytoskeleton, nuclear speck, perinuclear region of cytoplasm, spliceosome complex, nuclear inner membrane, nuclear matrix, brush border membrane, desmosome, and mRNA cleavage factor complex. As for molecular functions, the top 10 terms included RNA binding, DNA binding, guanyl-nucleotide exchange factor activity, protein kinase binding, mRNA binding, transcription corepressor activity, protein-macromolecule adaptor activity, telomeric DNA binding, RNA strand annealing activity, and pre-mRNA three-splice site binding.

Additionally, to further understand the enrichment of signaling pathways of the significantly downregulated proteins, the KEGG pathway enrichment was performed. The top 20 pathways were tight junction, regulation of actin cytoskeleton, autophagy, adherens junction, notch signaling pathway, spliceosome, RNA transport, mRNA surveillance pathway, SNARE interaction, proteoglycans in cancer, microRNAs in cancer, etc. (Fig. 4B). Furthermore, the protein-protein interactions involved in the several major enriched pathways were analyzed as shown in Fig. 4C. These data demonstrated that multiple cellular processes were regulated by PRV infection via downregulating the phosphorylation of cellular proteins.

### Multiple phosphoproteins are the potential anti-PRV targets

**Fig 5:**
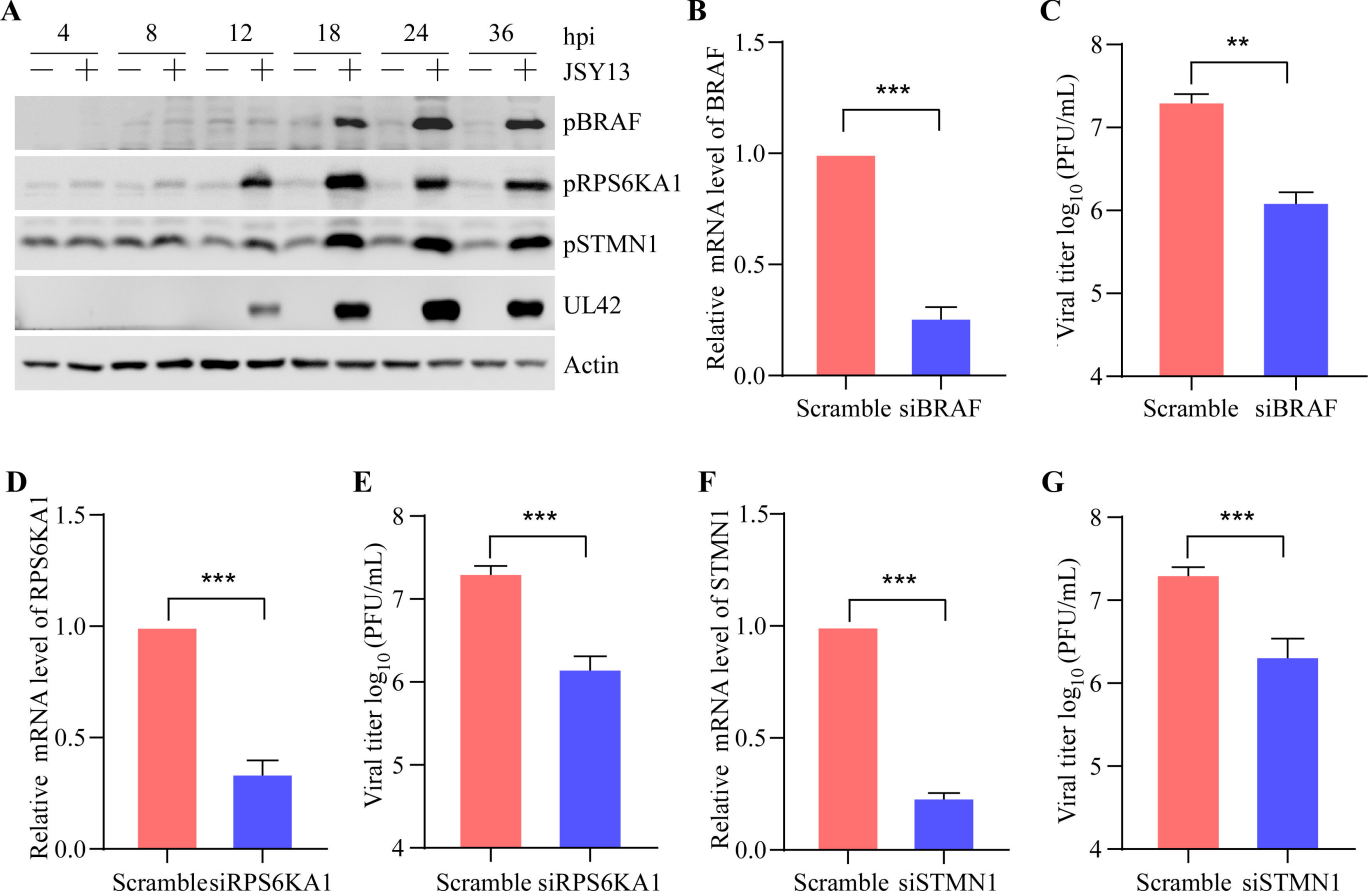
Multiple phosphoproteins are the potential anti-PRV targets. (**A**) PK15 cells were infected with JSY13 (0.5 MOI) for 4, 8, 12, 18, 24, and 36 hours. The cells were collected and subjected to western blot to verify the level of pBRAF, pRPS6KA1, and pSTMN1 for the identification of phosphoproteome. (**B**) PK15 cells in 6-well plates were transfected with siRNA against BRAF; 48 hours later, the cells were collected, and the mRNA level of BRAF was measured using QRT-PCR. (**C**) PK15 cells in 6-well plates were transfected with siRNA against BRAF; 36 hours later, the cells were infected with JSY13 (0.5 MOI) for 18 hours, then the cells were collected, and the viral titer was measured using plaque formation assay. (**D and E**) The experiments were performed as in panels **B** and **C**, respectively, except the siRNA against RPS6KA1 was transfected. (**F and G**) The experiments were performed as in panels **B** and **C**, respectively, except the siRNA against STMN1 was transfected. Significance is indicated as follows: *, *P* < 0.05; **, *P* < 0.01; and ***, *P* < 0.001.

To verify the results of the phosphoproteome, the upregulated phosphorylated proteins, including BRAF (FC = 2.37), RPS6KA1 (FC = 1.65), and STMN1 (FC = 3.16), were chosen for phosphorylation-level evaluation. PK15 cells were infected with PRV (0.5 MOI) for 4, 8, 12, 18, 24, and 36 hours, the infected samples were subjected to western blot analysis, and the results showed that BRAF, RPS6KA1, and STMN1 were all significantly upregulated during the infection of PRV, which demonstrated the reliability of our phosphoproteome (Fig. 5A). Phosphorylation is an important kinase-regulated process, and many kinds of cellular functions are regulated by phosphorylation. In this case, phosphoproteome might be a useful tool to find the restriction cellular factors involved in PRV replication. In order to find more restriction factors involved in PRV replication, the BRAF, RPS6KA1, and STMN1 were chosen for analysis of their role in PRV replication by knocking down them using siRNA. The effect of the siRNA on knockdown of target proteins was evaluated by detecting the mRNA of target genes. The QRT-PCR results showed that the siRNA could significantly downregulate the transcription of BRAF (Fig. 5B), RPS6KA1 (Fig. 5D), and STMN1 (Fig. 5F). Then, the role of them in PRV replication was evaluated using plaque formation assay. It was found that after the knockdown of BRAF (Fig. 5C), RPS6KA1 (Fig. 5E), and STMN1 (Fig. 5G), the replication of PRV was all significantly inhibited. These findings demonstrated that phosphoproteome might be a useful tool to identify the cellular factors involved in PRV replication.

## DISCUSSION

Phosphorylation is an important posttranslation modification that not only regulates the functions of the phosphorylated proteins themselves but also their downstream factors. In this study, a TMT-based phosphoproteome was conducted, and many kinds of phosphorylated proteins were detected. Furthermore, the characteristics and their interaction network were analyzed. This study provides a comprehensive survey of quantified phosphorylation events triggered by the PRV infection.

Previously, a phosphoproteome upon PRV infection was performed at 2.5 hpi at an MOI of 10, which demonstrated ATF2 was required for efficient PRV replication (27). Since one complete replication cycle of PRV typically ranges from 4 to 5 hours, which may vary depending on the type of cells, the 2.5 hpi just represented the early infection of PRV, during which the synthesis of the late genes might not be completed (4). In this case, the proteins phosphorylated by PRV-encoded protein kinases US3 and UL13 might not be detected. In this study, the time of infection was chosen based on the change of cell morphology and IFA experiment, which showed that nearly all cells were infected with PRV (0.5 MOI) at 18 hpi (Fig. 1C). At this time point, the abundance of phosphorylated proteins in the phosphoproteome might be greater than at 2.5 hpi. The result of our phosphoproteome analysis also supported this speculation, as a total of 7,289 phosphopeptides originating from 2,747 proteins were identified in our study, compared to 5,723 phosphopeptides originating from 2,180 proteins from the previous study. Except for host cellular proteins, a total of 50 potential phosphorylated PRV-encoded proteins were found to be phosphorylated during the viral infection (Table S2), which account for about 70% of PRV-encoded proteins. However, the phosphorylation events happened in viral proteins and their role in PRV replication still need to be confirmed.

The identification of the host cellular response is important for research on the study of virulence, replication capacity, cell tropism, and pathogenesis of the PRV (5, 28, 29). In addition, uncovering the interactions or links between viral replication and host cellular proteins will help us gain deep insight into the key cellular factors involved in viral replication. The main functions of the phosphorylated proteins screened in our study were subjected to GO and KEGG analysis. The results showed that multiple kinds of cellular responses were regulated by the phosphorylated proteins upon PRV infection, including autophagy, insulin signaling pathway, regulation of transcription elongation, stress granule assembly, and mRNA processing body assembly. All of these data demonstrated that PRV infection can manipulate multiple cellular pathways.

An important aim of the phosphoproteome is to reveal the role of the screened phosphorylated proteins in the replication of viruses, in order to identify the mechanisms of viral replication and contribute to the development of antiviral drugs (30 – 32). In this study, multiple important phosphorylated proteins that were involved in different cellular pathways were chosen for phosphorylation-level detection and analysis of their role in PRV replication. The immunoblotting results showed that the phosphorylation level of all three chosen proteins, including BRAF, RPS6KA1, and STMN1, were upregulated during PRV infection, which was consistent with the data in phosphoproteome. Additionally, an siRNA-based experiment showed that all these three proteins were necessary for efficient replication of PRV (Fig. 5B through G). Though just three proteins were chosen in this study, we have certain reason to believe that there must be many other phosphorylated proteins that participate in the replication of PRV. Therefore, the phosphoproteome not only provides a comprehensive insight into the interaction between the viral infection and host cellular responses but also can serve as a useful tool to find antiviral targets.

Although a total of 7,342 phosphosites with high confidence were screened out in this study, there were still other potential phosphorylated targets that were not detected in this phosphoproteome. For instance, it has been demonstrated that PRV infection can upregulate the phosphorylation of ATM, an important kinase in the regulation of DNA damage response, to promote PRV replication (18). Additionally, it was found that PRV infection could increase the phosphorylation of RIPK3 to regulate necroptosis (14). Moreover, it has been found that PRV infection can induce the phosphorylation of ERK1/2 to trigger an anti-apoptotic response (33). However, all these proteins, including ATM, RIPK3, and ERK1/2, were not screened out in this study, which might be due to the low amount or the collection time that was not ideal for certain proteins. Nonetheless, there are many phosphorylated proteins with the phosphorylated sites that are not reported anywhere before, the kinases in response to this phosphorylation, and their molecular functions are still worth further exploration.

In conclusion, a phosphoproteome was performed in this study, which demonstrates that PRV infection could trigger the phosphorylation of multiple proteins. The information obtained provides valuable insight into the manipulation of host signaling pathways upon PRV infection, laying a foundation for studying the mechanisms involved in PRV infection and designing of therapeutic strategies.

## MATERIALS AND METHODS

### Cells and virus

PK15 cells and Vero cells purchased from ATCC were grown in Dulbecco’s modified Eagle’s medium with 10% fetal bovine serum in a 5% CO_2_ incubator at 37°C. PRV JSY13 strain (MT157263.1) was isolated from the brain of a pig by our lab.

### Antibodies and reagents

Mouse anti-PRV-UL42 polyclonal antibodies were generated in our lab. Rabbit anti-pSTMN1 was purchased from Cell Signaling Technology (Beverly, MA, USA). Rabbit anti-BRAF and Rabbit anti-RPS6KA1 were purchased from Abclonal (Wuhan, China). Rabbit anti-actin was purchased from Proteintech (Wuhan, China). Alexa 555-conjugated goat anti-mouse IgG antibodies were purchased from Millipore (Billerica, MA, USA).

### Western blot analysis

PK15 cells were infected with JSY13 (0.5 MOI) for 4, 8, 12, 18, 24, and 36 hours. Then, the cells were washed with phosphate-buffered saline (PBS), collected using 2 × SDS sample buffer, and subjected to immunoblotting. For immunoblotting, the samples were separated by SDS-PAGE and transferred to a polyvinylidene difluoride membrane (Pall Cor, USA) at 200 mA for 2 hours. The membrane was blocked in 5% skim milk in PBS with 0.02% Tween 20 for 1 hour. Then, the membrane was incubated with primary antibodies at 4°C overnight and HRP-labeled secondary antibodies.

### Indirect immunofluorescence assay

PK15 cells were grown on coverslips overnight and then infected with JSY13 at an MOI of 0.5 for 4, 8, 12, 18, and 24 hours, respectively. Then, the cells were fixed with 4% formaldehyde and 0.1% Triton X-100 for 30 minutes. After washing with glycine-PBS three times, the slides were blocked with 3% BSA in PBS for 1 hour. Subsequently, the cells were incubated with mouse-anti-UL42 polyclonal antibodies (1:1,000) for 1 hour, followed by incubation with a secondary antibody (1:500) for 30 minutes. Nuclei were stained with DAPI, and the images were acquired with a Nikon fluorescence microscope (TS100-F; DSRi2).

### siRNA transfection

The PK15 cells in 6-well plates with 40% density were transfected with 50 nm siRNA against BRAF, RPS6KA1, and STMN1 using lipofectamine 3000 (Invitrogen), respectively. The control group was transfected with an equal amount of scramble siRNA. And 48 hours later, the cells were collected, and the mRNA levels of BRAF, RPS6KA1, and STMN1 were detected using QRT-PCR. For viral replication assay, after the siRNA was transfected for 36 hours, the cells were infected with JSY13 (0.5 MOI) for 18 hours, then the viral titer was measured using plaque formation assay in Vero cells as previously described (34). The sequences of the siRNA used in this study were listed below: siRNA-BRAF, GTGTGTTAATTATGATCAACT; siRNA-RPS6KA1, GCAACATCCTGTATGTTGACG; and siRNA-STMN1, GAAGAAGTGCGGAAGAACAAA.

### QRT-PCR

The total RNA of the cells was extracted using the RNA Extraction Kit (CwBiotech, Suzhou, China). Then the RNA was reverse transcribed into cDNA using the reverse transcription reagents EasyScript Reverse Transcriptase [M-MLV, RNaseH-] (Transgen, Beijing, China). The PRV mRNA level was evaluated by detecting its UL42 gene. Fluorescent QRT-PCR qMix (Vazyme, Nanjing, China) was used according to the manufacturer’s recommendations. The method in evaluation of the fold changes of mRNA level was the 2^-ΔΔCT^. The primers used in this study are listed in Table 1.

**TABLE 1:** The primer sequences for QRT-PCR

Name	Sequence
STMN1-F	5′-GTCCCATGAAGCTGAGGTCT-3′
STMN1-R	5′-CTTGTCCTTCTCCCGCAAAC-3′
RPS6KA1 -F	5′-AGCCCAGCAACATCCTGTAT-3′
RPS6KA1 -R	5′-AAGGTGTCATGAGAAGCCCA-3′
BRAF-F	5′-ACCACCCAATACCACAGGAG-3′
BRAF-R	5′-CCGGTCTCTCTGTCCAAACT-3′
GAPDH-F	5′-CCTTCATTGACCTCCACTACA-3′
GAPDH-R	5′-GATGGCCTTTCCATTGATGAC-3′

### Collection of PRV-infected samples for TMT-based LC-MS/MS

Three single-layer PK15 cells in 10 × cm cell culture dishes were infected with JSY13 (0.5 MOI) for 18 hours, the supernatant was discarded, and the cells were digested and centrifuged at 2,000 rpm for 5 minutes. Then the cells were washed with cold PBS three times and put in liquid nitrogen.

### Protein extraction and concentration determination

The collected samples were lysed with 300 µL of lysis buffer (2% SDS in 100 mM triethyl ammonium bicarbonate buffer) containing protease and phosphatase inhibitors. Next, the cell lysates were subjected to sonication for lysis. After sonication, the samples were centrifuged at 12,000 rpm for 20 minutes to remove insoluble fractions. Protein concentration was determined using a BCA Assay Kit (Thermo Fisher Scientific, USA) as described before (35). Then, 10 µg of protein from each sample was collected and separated using SDS-PAGE. The gel was stained by Coomassie brilliant blue overnight. Afterward, the gel was washed with water until the bands were clearly visualized and scanned using a digital gel image analysis system (Tanon 1600, China).

### Trypsin digestion and TMT labeling

Based on the result of protein quantification, 100 µg protein from each group was subjected to the reducing buffer (10 mM dithiothreitol, 8 M urea, 0.1 M triethylammonium bicarbonate [TEAB, pH 8.0]) in an ultrafiltration tube. The solution was incubated for 1 hour at 60°C. Next, IAA was added to the solution in the dark at room temperature for 40 minutes. Then, the solutions were centrifuged at 12,000 rpm for 20 minutes, and the flow-through solution was discarded. TEAB was added to the solutions and centrifuged at 12,000 rpm for 20 minutes. After washing three times, the filter units were transferred into new collection tubes, and TEAB was added and followed with sequencing-grade trypsin. The solutions were then incubated for digestion at 37°C for 12 hours. Finally, the collections of digested peptides were centrifuged and TEAB was added, followed by another round of centrifugation. Then, the lyophilized samples were resuspended in TEAB and 40 µL of each sample was transferred into new tubes for labeling. 88 µL of acetonitrile was added to TMT reagent. The centrifuged reagents were dissolved for 5 minutes, followed by mixing and another round of centrifugation. Next, the TMT label reagent was added to each sample for mixing and incubated at room temperature for 1 hour. Finally, 8 µL of 5% hydroxylamine was added to each sample and incubated for 15 minutes to stop the reaction. The labeled peptide solutions were lyophilized and stored at −70°C.

### Phosphopeptides enrichment

The phosphopeptides were enriched from samples using titanium dioxide beads (TiO_2_) as described before (36). Briefly, the labeled peptides were centrifuged at 12,000 rpm for 3 minutes at room temperature to precipitate peptides. Next, the phosphopeptides were resolved into the enrichment loading buffer (glutamate saturated solution with 2% TFA and 60% ACN). After vortexing for 15 minutes, the peptides were centrifuged. Then, a resolution of TiO_2_ beads (protein:TiO_2_ = 1:4, mass/mass) was added and vortexed for 15 minutes at room temperature. The sediment was collected after centrifugation at 6,000 rpm for 1 minute at room temperature. Then, 400 µL wash buffer (0.5% TFA/50% ACN) was added to the sediment, incubated for 15 minutes, and the samples were centrifuged at 6,000 rpm for 1 minute. The sediment was collected, and the 100 µL 10% NH_3_·H_2_O was used to wash off the phosphopeptides. After 15 minutes of incubation, the samples were centrifuged at 8,000 rpm for 3 minutes at room temperature. The supernatant was collected and then centrifuged at 12,000 rpm for 5 minutes. Finally, the phosphorylated peptide solutions were dried.

### LC-MS/MS and TMT data acquisition

The enriched phosphorylated peptides were analyzed using a Q-Exactive mass spectrometer (Thermo Fisher Scientific) equipped with a Nanospray Flex source (Thermo Fisher Scientific). The samples were loaded and separated by a C18 column on an EASY-nLCTM 1200 system (Thermo Fisher Scientific). The full scan was performed in the mass range of 300–1,500 *m*/*z* with a mass resolution of 70,000. The 10 most intense peaks in MS were fragmented with higher-energy collisional dissociation with normalized collisional energy (NCE) of 32. MS/MS spectra were obtained with a resolution of 17,500 with a maximum injection time of 80 ms. The Q-E dynamic exclusion was set for 30 s and run under positive mode.

### Database search and data analysis

The mass spectrometric data were queried against the UniProt database (*Sus scrofa* [Swine/Wild boar/*S. scrofa*/Pig] [9823]) and the PRV JSY13 strain (MT157263.1). Trypsin/P was selected as enzyme digestion mode, and cysteine alkylation was designed as the fixed modification in the database search. The quantitative method was set as TMT-6plex with a specified false discovery rate of 0.01. The significant fold change threshold was set at 1.5-fold.

### Bioinformatic analysis

The functional annotation of phosphorylated proteins was characterized using UniProt (http://www.uniprot.org/) (37) and GO (http://www.geneontology.org/). Protein functions were classified based on MapMan ontology, and the subcellular location was predicted from the consensus location available from SUBA3 (38). Phosphorylation motifs of the phosphorylated sites were analyzed by using the Motif-X algorithm in MoMo Modification Motifs (MEME Suite5.1.0, https://meme-suite.org/meme/tools/momo) (39). The KEGG analysis was performed by analyzing the candidate proteins using the STRING database (https://cn.string-db.org/), and the ClueGO plug-in of Cytoscape (Version: 3.7.2) was used for visualization as reported (40).

### Statistical analysis

Statistical significance between different groups was determined using Student’s *t*-test in GraphPad Prism version 8 (La Jolla, CA, USA). All experiments were performed at least three times, and the data are presented as means ± standard deviations.

## Data Availability

The raw files have been deposited to the ProteomeXchange (http://proteomecentral.proteomexchange.org) with the data set identifier PXD044682.

## SUPPLEMENTAL MATERIAL

Table S1 spectrum.03010-23-s0001.xlsx The total confident and significantly changed phosphosites.

Table S2 spectrum.03010-23-s0002.docx Potential phosphorylated PRV proteins.

ASM does not own the copyrights to Supplemental Material that may be linked to, or accessed through, an article. The authors have granted ASM a non-exclusive, world-wide license to publish the Supplemental Material files. Please contact the corresponding author directly for reuse.
