# Supplementary material for: Phosphoproteomic landscape of pseudorabies virus infection reveals multiple potential antiviral targets
Source: Microbiol Spectr. 2023 Nov 22;12(1):e03010-23. doi: 10.1128/spectrum.03010-23 (PMC10783065; doi:10.1128/spectrum.03010-23)
Supplement: Table S2 — Potential phosphorylated PRV proteins. [file spectrum.03010-23-s0002.docx]

**Table S2: Potential phosphorylated PRV Proteins. Totally 50 potential phosphorylated PRV encoded proteins were listed.**

| **Potential phosphorylated PRV Proteins** | | |
| --- | --- | --- |
| ORF-1 | UL35 | UL13 |
| UL54 | UL36 | UL12 |
| UL51 | UL37 | UL10 |
| UL52 | UL38 | UL9 |
| UL50 | UL39 | UL8 |
| UL49.5 | UL42 | UL6 |
| UL49 | UL44 | UL3.5 |
| UL48 | UL26 | UL3 |
| UL47 | UL25 | EP0 |
| UL46 | UL23 | IE180 |
| UL27 | UL22 | US1 |
| UL28 | UL21 | US2 |
| UL29 | UL19 | US3 |
| UL30 | UL18 | US7 |
| UL31 | UL17 | US8 |
| UL32 | UL16 | US9 |
| UL34 | UL15 |  |
